# Acromioclavicular and sternoclavicular joint dislocations indicate severe concomitant thoracic and upper extremity injuries in severely injured patients

**DOI:** 10.1038/s41598-020-78754-9

**Published:** 2020-12-10

**Authors:** M. Sinan Bakir, Rolf Lefering, Lyubomir Haralambiev, Simon Kim, Axel Ekkernkamp, Denis Gümbel, Stefan Schulz-Drost

**Affiliations:** 1 Department of Trauma and Reconstructive Surgery and Rehabilitative Medicine, Medical University Greifswald, Ferdinand-Sauerbruch-Straße, 17475 Greifswald, Germany; 2 Department of Trauma Surgery and Orthopedics, BG Hospital Unfallkrankenhaus Berlin gGmbH, Warener Straße 7, 12683 Berlin, Germany; 3 Faculty of Health, IFOM – Institute for Research in Operative Medicine, Witten/Herdecke University, Ostmerheimer Str.200, Haus 38, 51109 Cologne, Germany; 4 Department of Trauma and Orthopedic Surgery, University Hospital Erlangen, Krankenhausstr. 12, 91054 Erlangen, Germany; 5 Department of Trauma Surgery, Helios Hospital Schwerin, Wismarsche Str. 393-397, 19049 Schwerin, Germany

**Keywords:** Medical research, Risk factors

## Abstract

Preliminary studies show that clavicle fractures (CF) are known as an indicator in the severely injured for overall injury severity that are associated with relevant concomitant injuries in the thorax and upper extremity. In this regard, little data is available for the rarer injuries of the sternoclavicular and acromioclavicular joints (SCJ and ACJ, respectively). Our study will answer whether clavicular joint injuries (CJI), by analogy, have a similar relevance for the severely injured. We performed an analysis from the TraumaRegister DGU (TR-DGU). The inclusion criterion was an Injury Severity Score (ISS) of at least 16. In the TR-DGU, the CJI were registered as one entity. The CJI group was compared with the CF and control groups (those without any clavicular injuries). Concomitant injuries were distinguished using the Abbreviated Injury Scale according to their severity. The inclusion criteria were met by n = 114,595 patients. In the case of CJI, n = 1228 patients (1.1%) were found to be less severely injured than the controls in terms of overall injury severity. Compared to the CF group (n = 12,030; 10.5%) with higher ISS than the controls, CJI cannot be assumed as an indicator for a more severe trauma; however, CF can. Concomitant injuries were more common for severe thoracic and moderate upper extremity injuries than other body parts for CJI. This finding confirms our hypothesis that CJI could be an indicator of further specific severe concomitant injuries. Despite the rather lower relevance of the CJI in the cohort of severely injured with regard to the overall injury severity, these injuries have their importance in relation to the indicator effect for thoracic concomitant injuries and concomitant injuries of the upper extremity. A limitation is the collective registration of SCJ and ACJ injuries as one entity in the TR-DGU. A distorted picture of the CJI in favor of ACJ injuries could arise from the significantly higher incidence of the ACJ dislocation compared to the SCJ. Therefore, these two injury entities should be recorded separately in the future, and prospective studies should be carried out in order to derive a standardized treatment strategy for the care of severely injured with the respective CJI.

## Introduction

The clavicle has a particular importance due to its function as a connection from the upper extremity to the trunk^1^. Since the impact of the frequently occurring clavicle fractures (CF) in severely injured patients has been thoroughly investigated, minimal data are available on the comparatively rare injuries of the sternoclavicular and acromioclavicular joints (SCJ and ACJ, respectively)^2–9^. CFs are known as indicators of further injuries (thoracic and upper extremity injuries) in severely injured patients and are associated with an increased overall injury severity^6,8^ and have been described in an analysis of data from the TraumaRegister DGU (TR-DGU)^6,8,10^. Therefore, the clavicle can be described as a kind of “gatekeeper of the thorax”^11^. The question of whether clavicular joint injuries (CJI) show similar relationships, which are analogous to CFs, has not been answered yet. Some case reports/case studies indicate such analogies exist^3,12^. Particularly, in the case of dorsal SCJ dislocation, the anatomical proximity can lead to severe concomitant injuries to neurovascular structures in the area between the thorax and the arms. These injuries are often caused by a severe trauma mechanism that could hypothetically be associated with a poorer outcome^13–15^.

Therefore, our primary hypotheses are that injuries of the SCJ or ACJ could be assumed, equivalent to CFs, to be an indicator for severe trauma with increased overall injury severity, and to be associated with relevant concomitant injuries of the thorax and the upper extremities^6,8^.

As secondary endpoints, we expect the trauma mechanism to be stronger than in the control group^7^. We assume that in addition to the increased Injury Severity Score (ISS), this trauma could also have a negative impact on the outcome as represented by the length of stay in the intensive care unit (ICU) and in the hospital^3^. Since the CJIs mostly do not represent life-threatening injuries, and deaths are extremely rare, we do not expect any relevant difference in mortality^16^. In comparison to previous results and to the group of patients with a CF without SCJ/ACJ involvement, we would expect similar results for the CJI group^6^. Since CJIs are not reflected in the primary relevant airway, breathing, circulation, disability, and exposure (ABCDE) problems, CJIs can potentially be missed in emergency situations involving the severely injured population. Therefore, we also hypothesize that CJIs in polytrauma or severely injured patients often undergo delayed diagnoses.

Our aim was to show the importance of CJI for the severely injured. The question of whether the injury entities to the SCJ and ACJ, similar to CF, are indicators of a severe trauma and/or severe concomitant injuries, indicating that these patients require special attention or whether CJI are merely a side issue that have no impact on the outcome of the severely injured, is discussed in this study.

## Materials and methods

### TraumaRegister DGU

The TraumaRegister DGU of the German Trauma Society (Deutsche Gesellschaft für Unfallchirurgie, DGU) was founded in 1993^17^. The aim of this multi-centre database is a pseudonymised and standardised documentation of severely injured patients. Data are collected prospectively in four consecutive time phases from the site of the accident until discharge from hospital: (A) Pre-hospital phase, (B) Emergency room and initial surgery, (C) Intensive care unit and (D) Discharge. The documentation includes detailed information on demographics, injury pattern, comorbidities, pre- and in-hospital management, course on intensive care unit, relevant laboratory findings including data on transfusion and outcome of each individual. The inclusion criterion is admission to hospital via emergency room with subsequent ICU/ICM care or reach the hospital with vital signs and die before admission to ICU. The infrastructure for documentation, data management, and data analysis is provided by AUC—Academy for Trauma Surgery (AUC—Akademie der Unfallchirurgie GmbH), a company affiliated to the German Trauma Society. The scientific leadership is provided by the Committee on Emergency Medicine, Intensive Care and Trauma Management (Sektion NIS) of the German Trauma Society. The participating hospitals submit their data pseudonymised into a central database via a web-based application. Scientific data analysis is approved according to a peer review procedure laid down in the publication guideline of TraumaRegister DGU.

The participating hospitals are primarily located in Germany (90%), but a rising number of hospitals of other countries contribute data as well (at the moment from Austria, Belgium, China, Finland, Luxembourg, Slovenia, Switzerland, The Netherlands, and the United Arab Emirates). Currently, approx. 33,000 cases from more than 650 hospitals are entered into the database per year. Participation in TraumaRegister DGU is voluntary. For hospitals associated with TraumaNetzwerk DGU, Participation in TraumaRegister DGU is voluntary. For hospitals associated with TraumaNetzwerk DGU, however, the entry of at least a basic data set is obligatory for reasons of quality assurance.

The present study is in line with the publication guidelines of the TraumaRegister DGU and registered as TR-DGU project ID 2018-020. All patients, their parents or a legal guardian gave their informed written consent in collecting and publishing data. All data were collected anonymously and information about publication of data were disclosed to all participants. Additional approval from local ethics committee was not necessary due to the retrospective evaluation, the informed consent, the existing ethics vote and the international character of the registry (Medical University Greifswald, ethics committee).

#### Patients

In order to achieve homogeneity of the datasets and to minimize variations related to the different rescue systems, only trauma patients who were treated in participating hospitals from Germany, Austria, and Switzerland were analyzed for our study. This analysis included n = 708 hospitals from 2008 to 2017. Early transfer out to another trauma center within 48 h was an exclusion criterion in order to prevent from double counting. Therefore only primary admitted and treated patients or patients transferred in later were analyzed. Patients of all ages were included in the study. Since the evaluation focused on severely injured patients, the study concentrated on patients with a minimum ISS of 16, which is the common definition of severely injured patients^18^. There were no further restrictions on the data record.

Due to feasibility reasons, TR-DGU did not use all 2000 Abbreviated Injury Scale (AIS) codes for documentation, but only about 400. Similar injuries with the same AIS severity level were merged for this purpose. Therefore, injuries of the SCJ and ACJ were coded together as one entity in the TR-DGU. The CJI were divided into two degrees of severity: (1) low-grade CJI in case of a sprain or subdislocation and (2) high-grade CJI in case of an open or displaced injury to the SCJ/ACJ. The group of the CJI was compared to the group of CF, and in addition to a control group without any injury of the clavicle or its related joints. In order to define the control group, patients with isolated head injuries were excluded, since these have to be considered specifically and could distort the results. Combination injuries of CJI and CF were considered. Anatomically, the clavicle was assigned to the arms when categorizing the body regions in the TR-DGU as the SCJ/ACJ was.

For the analysis of the main study focus, both the ISS and the New Injury Severity Score (NISS) were considered as parameters for the overall injury severity^18,19^. Concomitant injuries were differentiated according to the Abbreviated Injury Scale ([AIS] version 2005, Association for the Advancement of Automotive Medicine, Barrington, IL, USA): AIS ≥ 3 was defined as a serious concomitant injury^20^. The location was also examined separately. After the anatomical classification based on the TR-DGU, the injuries were assigned to the head, face, neck, thorax, abdomen, spine, extremities (arms, legs) and pelvic regions. In addition, concomitant injuries to the shoulder-girdle and the thoracic injuries were broken down further and considered separately. In the defined groups, we also reported data on the patient demographics (age and gender distribution), the injuries (pattern, severity, trauma mechanism, delayed detection of initially missed injuries) and the outcome (duration of the intubation, lengths of stay in the ICU and hospital, survival and predicted mortality using the RISC II score)^21,22^. Delayed detection was defined as detection of an injury after admission to ICU and therefore being initially missed during primary and secondary survey. For the evaluation of missed injuries, we included only patients with existing information of time of diagnosis detection. Additionally, we performed a sub-analysis regarding the number of related surgical treatments. In addition to the surgeries, the analysis includes interventions of all kinds in analgosedation/anesthesia, such as the closed reduction of a (sub)dislocated joint.

#### Statistics

Results were presented as mean with standard deviation (SD) in case of metric measurements, and percentages in case of categorical data. The median was also given for metric parameters in which the mean and the median differ noticeably. Due to the large sample size, formal statistical testing was avoided since even minor (and clinically non-relevant) differences would formally become statistically significant. A 95% confidence interval (95% CI) was calculated for selective rates. SPSS statistical software was used for analysis (IBM Inc., Armonk, NY, USA).

## Results

### Prevalence

**Figure 1:**
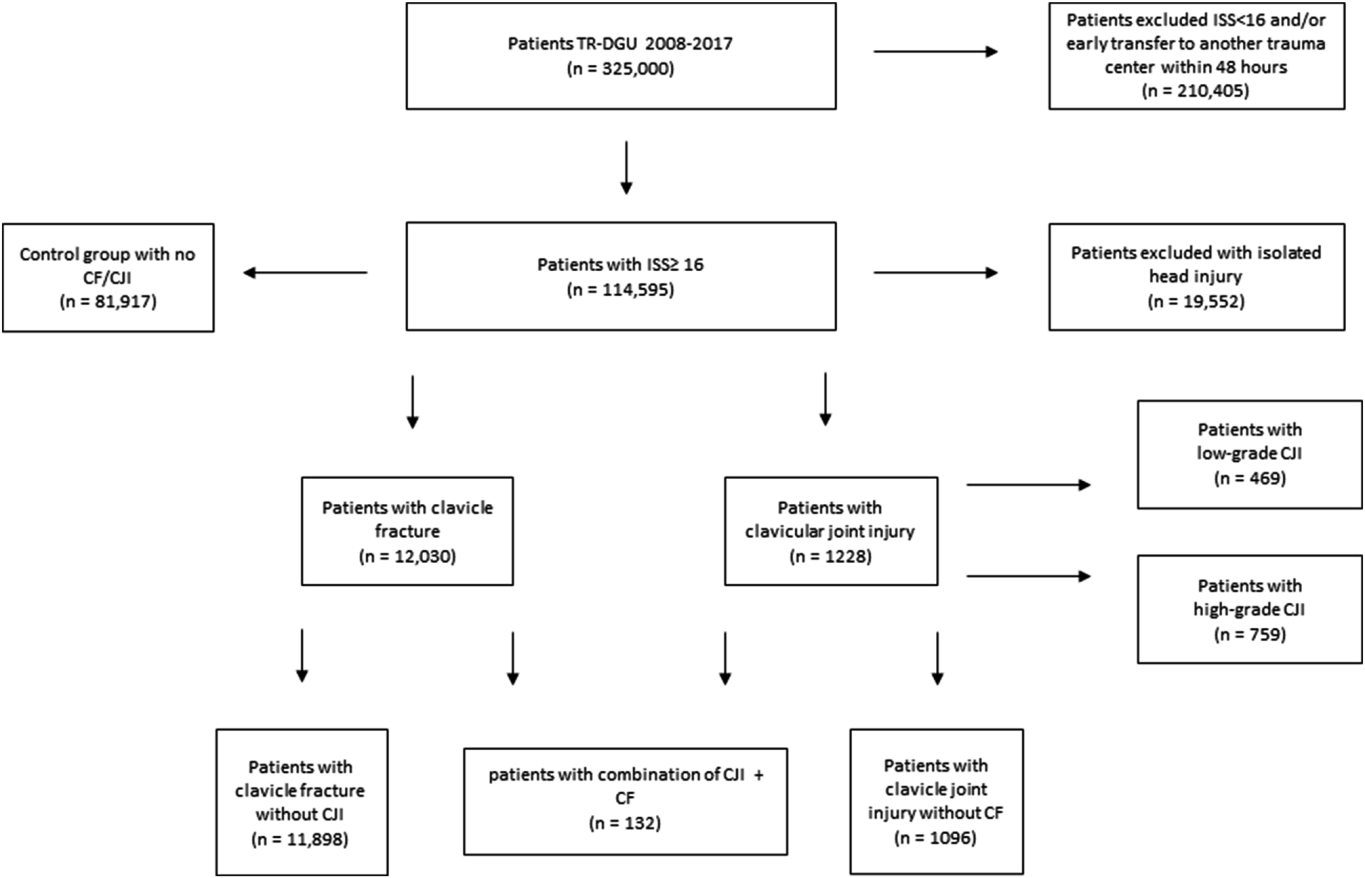
Flowchart for patient selection and incidence. The clavicular joint injuries (CJI) were divided into two degrees of severity: low-grade CJI for the purpose of a sprain/subdislocation and high-grade CJI with the meaning of an open or displaced injury. TR-DGU = TraumaRegister DGU; n = number; ISS = injury severity score; CJI = clavicular joint injury; CF = clavicle fracture.

The proportion of patients in the total cohort who suffered a CJI was 1.1% (95% CI 1.01–1.13) with 0.4% for low-grade CJI as a sprain/subdislocation and 0.7% for high-grade CJI as an open or displaced injury (Fig. 1). CF was significantly more common with 10.5% (95% CI 10.30–10.70) of all injuries in the cohort.

### Combination injuries

A total of 0.1% of the patients were affected by both injuries, so they suffered extremely rare clavicle-associated, ligament-osseous combination injuries (Fig. 1). With the focus on the CJI, however, this means that in the case of a CJI there is a high proportion of combination injuries since these are associated with a CF (AIS 2) in 10.7% of all CJI.

### Demographics

**Figure 2:**
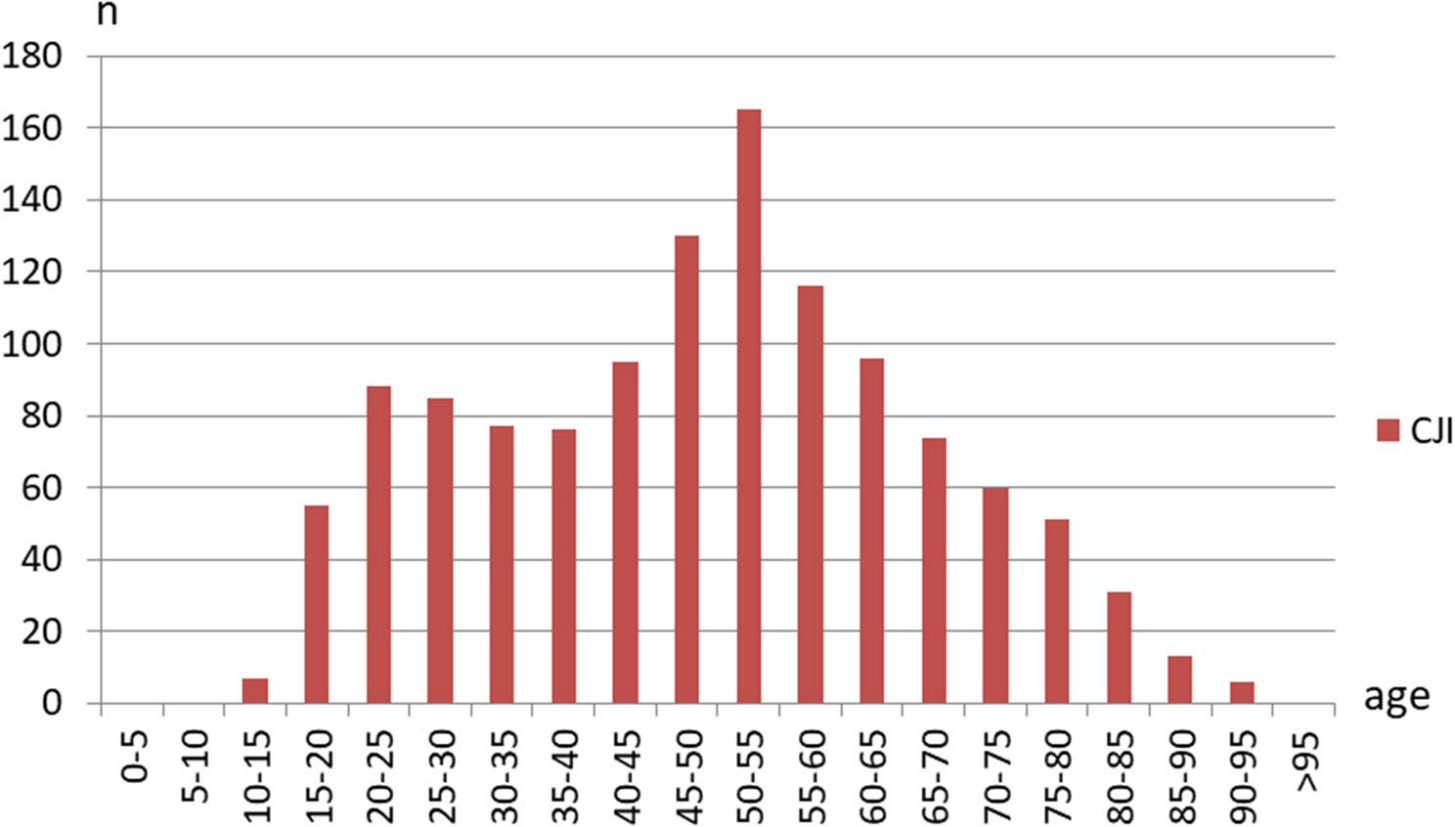
Age distribution of clavicular joint injuries. CJI = clavicular joint injury. n = number.

The age pattern for CJI showed a peak between 45 and 60 years. A second, considerably smaller peak appeared around the age of 20 years (Fig. 2). Incomplete data sets were available in three patients so that the average age for n = 1225 in the CJI group was slightly younger than for patients with CF and patients without CJI or CF (Table 1). Men were affected more frequently in CJI with 85.3%, which is noticeably more common than in the control group and in patients with CF (Table 1).

**Table 1:** Demographics and concomitant injuries AIS ≥ 2.

	CJI	CF	Control
Age (SD)	48.3 (18.0)	51.3 (20.4)	49.8 (21.7)
Male (%)	85.3	71.7	71.6
Head (%)	52.9	62.6	52.4
Face (%)	13.6	15.3	18.4
Neck (%)	1.3	1.7	1.8
Thorax (%)	84.2	86.4	70.7
Abdomen (%)	21.2	21.6	26.8
Spine (%)	37.5	38.6	39.9
Upper extremity (%)	78.0	100^a^	30.9
Lower extremity (%)	23.2	23.6	33.1
Pelvis (%)	19.0	20.8	27.7

All values of associated injuries are given as a percentage of all patients in the corresponding group. AIS = Abbreviated Injury Scale; SD = standard deviation; CJI = clavicular joint injury; CF = clavicle fracture; Control = control group with no CF/CJI.

^a^Per definition, a CF belongs to fractures of the upper extremity.

### Main results

#### ISS/NISS

Overall, the patients were found to be less severely injured than the controls in terms of overall injury severity in the case of CJI. The overall injury severity was assessed using the ISS/NISS. The average ISS as well as the NISS were below the average of patients the control group without any clavicle-related injury in terms of CJI and above the average for CF (Table 2).

**Table 2:** Outcome parameter concerning injury severity.

	CJI	CF	Control
ISS	25.7 (9.2)	28.1 (10.9)	27.6 (11.5)
NISS	30.9 (11.9)	33.4 (13.4)	32.8 (14.0)
Days intubated	4.5 (9.1) median 0	5.8 (10.0) median 1	4.9 (9.7) median 1
ICU stay (d)	9.0 (12.1) median 4	10.6 (12.5) median 6	9.5 (12.8) median 4
Hospital stay (d)	22.1 (18.4) median 17	22.0 (19.1) median 18	21.9 (22.0) median 16
Hospital stay, survivor only (d)	22.5 (18.4) median 18	23.7 (18.9) median 19	24.8 (22.2) median 19

All values are presented as a mean value (standard deviation). The median was added for parameters in which the mean and the median differ noticeably.

*ICU* intensive care unit, *CJI* clavicular joint injury, *CF* clavicle fracture, *Control* control group with no CF/CJI, *d* days.

### Concomitant injuries

**Figure 3:**
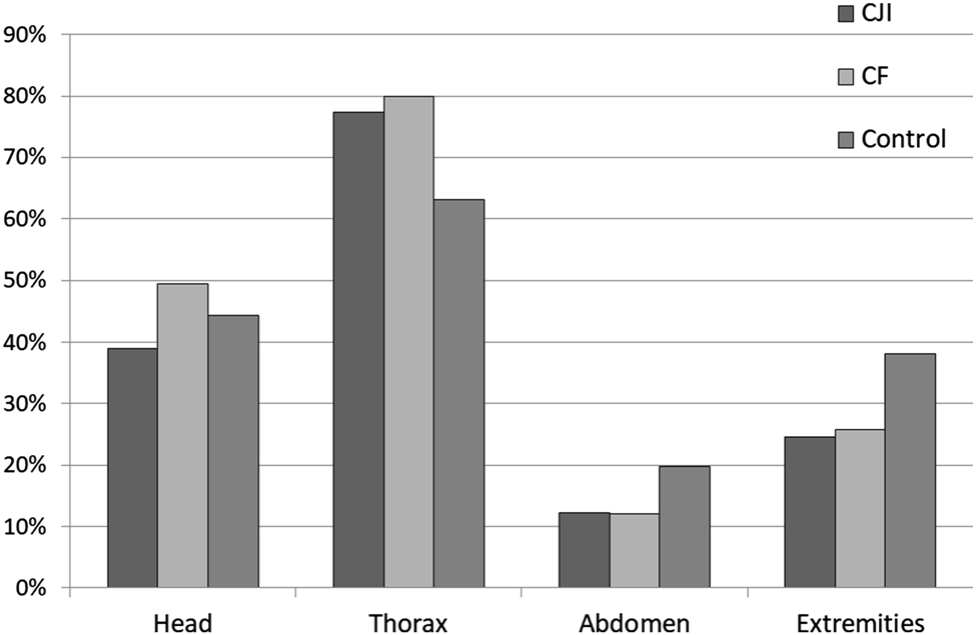
Serious concomitant injuries AIS ≥ 3, based on the classification according to the ISS body regions. Extremities: including shoulder and hip joint. AIS = Abbreviated Injury Scale; CJI = clavicular joint injury; CF = clavicle fracture; Control = control group with no CF/CJI.

With regard to serious concomitant injuries (AIS ≥ 3), severe thoracic injuries were more common for CJI (in addition to CF), while other body parts were less frequently affected by serious concomitant injuries (Fig. 3). Also considering the group of any concomitant injuries (AIS ≥ 2), there were disproportionately frequent concomitant injuries at the thorax and arms compared to the control group, while other anatomic regions were affected similarly (Table 1).

Regarding the concomitant thoracic injuries, CJI and CF were affected disproportionately often (Table 3); a hematothorax/pneumothorax was 1.6 times as likely in comparison to the control group and serial rib fractures (≥ AIS 3) 1.5–1.7 times. Regarding concomitant shoulder-girdle injuries, all entities were more common in CJI/CF, whereby especially concomitant injuries of the sternum and the subclavian artery/subclavian vein predominated in CJI and appeared more often than in CF (Table 4).

**Table 3:** Concomitant thoracic injuries.

	CJI	CF	Control
Hemato/pneumothorax	48.9	49.2	31.4
**Rib fracture according to AIS severity**			
AIS 1	5.4	4.5	4.2
AIS 2	4.7	5.4	4.5
AIS 3	41.5	44.0	27.1
AIS 4	10.8	12.4	5.6
AIS 5	5.5	6.5	5.0
None	32.1	27.3	53.7

All values are given as a percentage of all patients corresponding to the group. The rib fractures are sorted according to the AIS severity.

*CJI* clavicular joint injury, *CF* clavicle fracture, *Control* control group with no CF/CJI.

**Table 4:** Concomitant shoulder-girdle injuries.

		CJI	CF	Control
Sternum	(AIS 2)	11.6	9.6	7.9
Humerus	(AIS 2/3)	7.9	6.9	7.9
Scapula	(AIS 2)	18.8	21.5	8.0
Shoulder joint	(AIS 1/2)	3.2	0.8	1.2
A./V. subclavia	(AIS 3/4)	1.5	0.4	0.2

All values are given as a percentage of all patients corresponding to the group.

*CJI* clavicular joint injury, *CF* clavicle fracture, *Control* control group with no CF/CJI.

### Clinical parameters

#### Trauma mechanism

**Figure 4:**
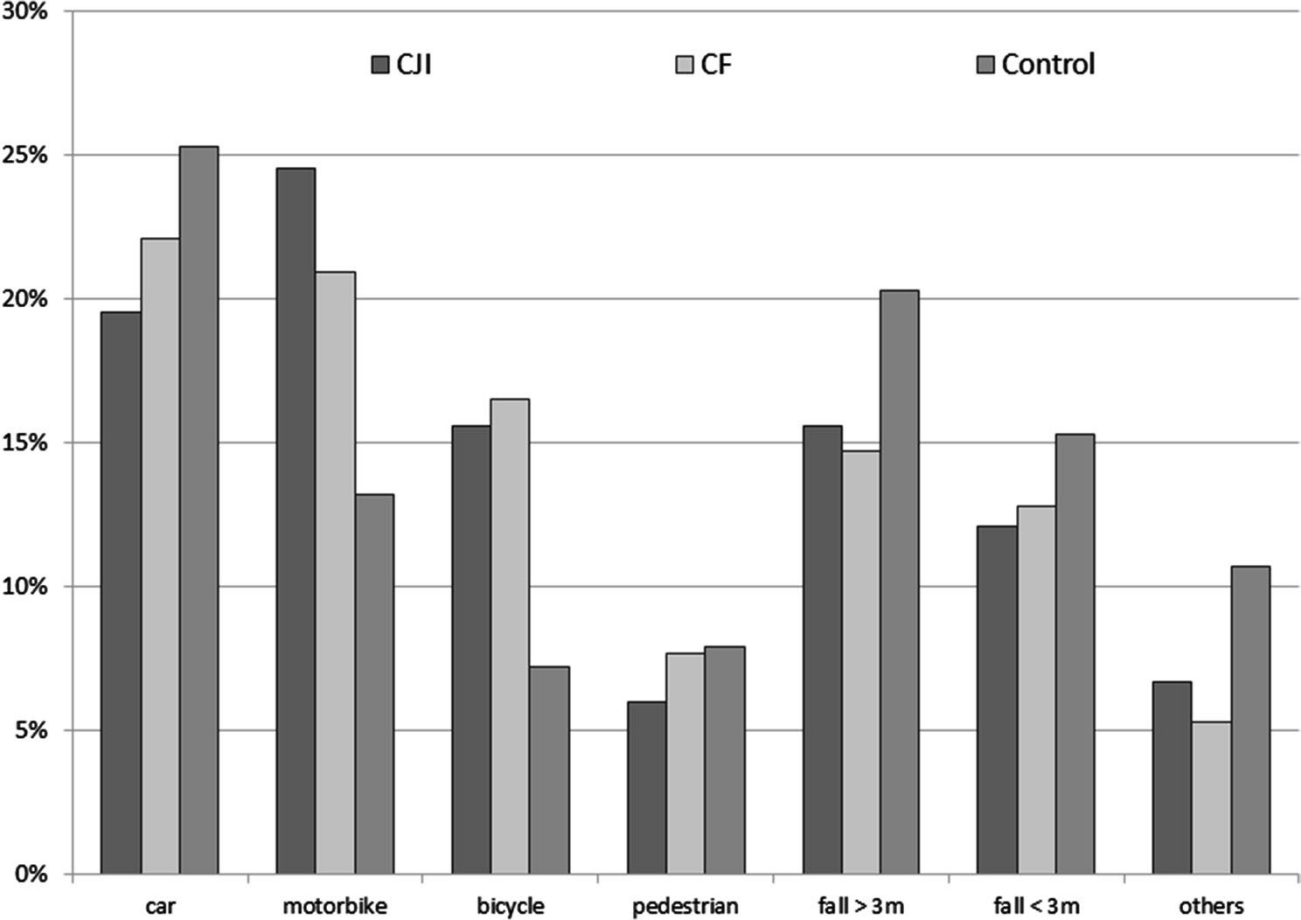
Trauma mechanism. CJI = clavicular joint injury; CF = clavicle fracture; Control = control group with no CF/CJI.

In almost all cases, a blunt trauma mechanism caused the injuries (approximately 99% for CJI and CF versus approximately 96% for the controls). A traffic-related accident was much more common in the CJI group and to a lesser extent, in the CF group in comparison to the control group. These were particularly due to two-wheeler accidents (traffic accidents by motorcycle/bicycle), which were far more often than in the controls (Fig. 4).

### Operative treatment

The sub-analysis regarding the number of a related surgical treatment included n = 870 patients with n = 325 and 545 low- and high-grade CJIs, respectively. While only 12.6% of the low-grade injuries were treated surgically, the amount of high-grade CJI was at 43.3% (in total 31.8%).

### Outcome

#### Injury severity

As already shown for ISS/NISS, the CJI group was less severely injured than the control group with respect to further collected parameters but with a longer hospital stay, while the CF group shows a higher injury severity (Table 2). In addition to the ISS/NISS, the injury severity was assessed based on the number of days in hospital, on ICU period, and the number of days the patient was intubated. The days with intubation and in the ICU also showed a similar tendency as reported for the ISS/NISS with a number of days for CJI below and for CF above the average of the control group. In contrast, the overall length of stay in the hospital was longer for CJI (and CF) than for the controls. Regarding only the hospital stay of the surviving patients, a reverse result with a shorter length of stay for CJI is shown. Since the mean and the median differ noticeably in the days with intubation and in the length of stay in the hospital and on ICU, the median was also given. The median between the different groups differs less, but shows a similar tendency as the mean.

#### Mortality

The mortality rate of the CJI was approximately one third of the value compared to the CF group (Table 5). However, both were low compared to patient mortality without any type of clavicular injury. The Revised Injury Severity Classification II (RISC II) for the predicted mortality was close to the actual mortality; for CJI and CF, the mortality was below the value of the RISC II, and for control group, it was above.

**Table 5:** Observed and expected mortality.

	CJI	CF	Control
Primary admitted patients	1107	10,584	72,964
Mortality	4.6%	12.2%	16.5%
Expected mortality based on RISC II	7.7%	13.6%	16.2%
Difference observed – expected mortality	− 3.1%	− 1.4%	+ 0.3%

Mortality and RISC II are given as a percentage of all primary admitted patients corresponding to the group. The number of primary admitted patients was added since only this group could be evaluated for comparison with the RISC II score.

*RISC* Revised Injury Severity Classification, *CJI* clavicular joint injury, *CF* clavicle fracture, *Control* control group with no CF/CJI.

### Missed injuries

In the case of low-grade CJI, 91.5% (n = 213) were diagnosed initially in the primary survey before transfer to the ICU. At high-grade CJI (n = 333), only a rate of 90.7% primarily diagnosed injuries was found. The overall rate of initially missed CJI was 9.0% (from n = 546).

## Discussion

### Prevalence

In addition to our major findings, we were able to demonstrate that the proportion of all patients without a higher-grade CJI was > 99% so that a severe injury in this area is an absolute exception for the severely injured. Among those affected, the age pattern showed its peak around the age of 50 years. This confirms previous studies in patients with shoulder-girdle injuries^9,23^. Since there is generally a similar age distribution for the severely injured, this does not differ from one another^24^. A second, lower peak describes the group of patients from 20 to 25 years, who is often associated with increased risk behavior and is considered to be particularly at risk for severe injuries, also in the area of the clavicle^9,24–26^.

### Main results

In this study, we have shown that the overall injury severity as measured with ISS and NISS was lower in patients with CJI than in the control group patients. Contrary to our hypothesis, CJI cannot be assumed as an indicator for a more severe trauma, in contrast to patients with CF in which the total injury severity is higher than the average. On the contrary, this propagated connection could be confirmed for CF^6^.

However, our hypothesis that the CJI could be an indicator of further specific severe concomitant injuries was confirmed. Thoracic injuries were clearly more common in the CJI group in both moderate and serious concomitant injuries. Due to the clavicle’s function as a connection between the upper extremity and the thorax, it is not surprising that injuries to the adjacent joints have, in particular, concomitant injuries in these proximate regions of the body. This is consistent with previous analyses^2,3^. This relationship was also shown in preliminary work on the CF, which we were able to confirm as well as that for CJI^2,6,8,10^. Severe thoracic injuries have often been detected in the past in patients with medial clavicle injuries such as SCJ dislocations^2^ Hence the denotation of the clavicle as the gatekeeper of the thorax is accurate^11^. This statement could also be affirmed when working on costoclavicular combination injuries^27^.

Overall, concomitant injuries in CJI were less or equally frequent compared to the control group with the exception of concomitant thoracic injuries. Looking closer at these thoracic injuries, rib fractures are common in CJI but even more common in CF. This may be due to the fact that the CJI are not separated after ACJ and SCJ dislocation. Due to the anatomic proximity, rib fractures are more likely to result from SCJ rather than from ACJ injuries^2^. However, since the incidence of ACJ injuries is also many times higher than that of SCJ injuries, this ratio in favor of ACJ injuries would lower the average probability of rib fractures in the CJI group^9^.This ratio would also explain the more common rib fracture as a concomitant injury in CF although hypothetically this should be more common in SCJ injuries than in CF.

Concerning the CJI/CF, all entities of concomitant shoulder-girdle injuries occurred more frequently than in controls, whereby concomitant injuries of the sternum and subclavian vessels predominated in the CJI and continued to appear obviously more than in the CF. This finding is most likely due to SCJ injuries. Sternal concomitant injuries in addition to neurovascular injuries at dorsal dislocations of the SCJ have been described, particularly in the case of higher-grade lesions of the SCJ^13^, which highlights the complexity of higher-grade SCJ injuries^2,3,28,29^.

Clavicular combination injuries of CJI, including a coincident CF, could be viewed as the most complex entity of CJI with CJI being more frequently associated with CF than in every 10^th^ case. These combination injuries are rare but have hardly been investigated and should be investigated further because of the difficulty in diagnoses^2,3,9,12,30–32^.

### Clinical parameters

In order to avoid missing injuries in the future, focus should be placed explicitly on the examination of the SCJ/ACJ, especially in the case of a (suspected) severe injury after a two-wheeler accident since this type of accident was the trauma mechanism in > 40% of CJI cases. We were able to confirm indications of this relationship from previous studies, but this study is the first time that has provided proof of the importance of two-wheeler accidents as a decisive factor for clavicular injuries^3,7,33^. As a trauma mechanism, it can be assumed that a lateral impact combined with jerky forward movement or a direct blow to the top of the shoulder led to the injury^4,34^. It can be assumed that the force impact at the CJI was slightly greater than at the CF as the number of motorcycle accidents was even more common^7^. Except for this difference, a similar distribution of the trauma mechanism was found with respect to CF.

The number of (surgical) interventions related to CJI in severely injured patients was less in comparison with previous work with an intervention rate of about 30% in our study. Current studies show that the rate of interventions for SCJ and ACJ dislocation is twice as high^2,35^. Based on a ratio of SCJ to ACJ dislocation of approximately 1 to 44, these interventions would result in a common surgery rate for CJI of approximately 60%^2,9^. This finding is more common than we demonstrated for the severely injured. A feasible reason might be the focus on more life-threatening injuries according to the damage-control concept and a later position in the priority pattern of multiple injuries^36–38^. Depending on individual circumstances, a severely injured and therefore, for some reason, inoperable patient might lead to the choice of a more conservative treatment despite a general indication for surgery. This finding coincides with results concerning CF cases, which were treated surgically four times as often in monotrauma compared to polytrauma patients^10^. The fact that increasing numbers of operations are performed with increasing injury severity is entirely plausible since low-grade CJIs are usually no indication for surgery^35,39^.

### Outcome

Overall, the patients showed less severe injuries than the control group in the case of CJI based on the various measured outcome parameters but with a longer hospital stay. Since the consequences of the injury are not life-threatening, a higher mortality in the controls could also lead to a shorter hospital stay since the highest mortality is in the first days of the inpatient stay^40^. This higher mortality bias would therefore be no indication of a less severe injury despite the shorter length of stay in the control group but would actually indicate the opposite. This is supported by the differentiated consideration of the hospital stay after excluding the deceased patients. The length of stay of the survivors thus confirmed the tendency that the CJI were less severely injured than the CFs and the controls. A shorter stay in the controls could also be due to early transfer to another hospital, for example, for neurological rehabilitation due to the more common occurrence of severe traumatic brain injury in the control group.

Due to the inclusion criterion of the TR-DGU that treatment in the ICU / intermediate care station after the emergency room treatment is necessary, all patients have a certain duration of days at ICU. Based on the inclusion criteria, it was also determined that only severely injured patients with an ISS of ≥ 16 were considered, which of course leads to a shift in patients with a longer length of stay in the ICU. Although CJIs are more affected by (severe) thoracic concomitant injuries, the average duration of intubation is shorter than in the controls. However, since the duration and frequency of intubation can also depend on the presence of a severe traumatic brain injury, the latter would be a possible explanation for this initially apparent contradiction^41^.

Considering the outcome parameters based on the median results, there was an analogical tendency as described. However, the median value was distinctly lower than the mean. Although the patients with CJI were intubated for an average of 4.5 days, the median of zero days shows that the majority of the patients have not been ventilated at all. Similarly, the length of stay in the hospital and the days on ICU were shorter. This suggests that particular outliers of patients with a very length of stay can falsify the interpretation, but the tendency between the groups remains the same.

The mortality in the CJI group confirms a lower injury severity compared to the control group. The mortality for the controls was approximately 3.6 times that of CJI. With a similar ISS/NISS, we attribute this large difference to severe traumatic brain injuries, which have a high mortality and are more common in the control group^41^. Since the CJI group also includes patients with low-grade CJI, some of which consist only of a distortion, the low mortality is attributable to the predominance of low-grade injuries. In our assumption, these low-grade injuries generally consist of a less severe injury pattern (including concomitant injuries) in contrast to high-grade CJI with a more severe trauma, not only in the area of the shoulder-girdle. Less traumatic brain injuries and the predominance of low-grade CJI could also be reason for the discrepancy in the difference between predicted and actual mortalities when comparing CJI with CF^41^. The patients with CJI died less often than predicted by the RISC II. Another potential explanation could be that certain deviations in the RISC II are to be expected, especially in the case of subgroups with smaller numbers of cases as in case of CJI^21,22^. The predicted mortality calculated with the RISC II was relatively close to the observed mortality for patients without clavicle-related injuries and those with CF.

### Missed injuries

Early diagnosis is also of long-term relevance. Although an SCJ injury has a significantly lower incidence compared to the CF, post-traumatic arthrosis can be expected in particular in cases of untreated higher-grade SCJ dislocation in addition to ACJ dislocations^9,42^. This type of arthrosis can lead to relevant functional limitations in the future even if this injury is of inferior importance in the primary survey of the severely injured. Due to the lack of consideration as life-threatening, missing these injuries in the primary survey is quite conceivable. Because of the low incidence shown, a routine examination that considers the injury entity as CJI, at least regarding the SCJ, is rather rare. A lack of routine examination could also be a reason why CJIs undergo delayed diagnoses and are overlooked primarily in about one in ten cases of such an injury. Preliminary studies have shown that deficient routine is a factor and that missed injuries are less in severely injured patients if they are treated by a trauma surgery specialist^43^. The rate of primarily missed injuries is higher than previously presented in the literature. This is the first study reporting the missed injury rate at CJI specifically, but there is a wide range of literature on missed injuries with a wide spread distribution of missed injuries and delayed diagnosis incidence rates from 1 to 39% depending on the localization of the injury^44^. Depending on the definition and severity of the missed injury, however, most of these can be seen in the spectrum from most likely 1% to 5% of all trauma patients^44–46^. In comparison to data from the same collective of the TR-DGU regarding missed injuries, the rate was low compared to missed foot injuries^47^. The hypothesis that higher-grade and more severe CJIs can be recognized more easily and thus also be diagnosed more easily could not be substantiated in our study. There was rather a slight difference to the disadvantage of the high-grade CJI in this regard. It is possible that in the latter case not only is the CJI more severe but also that other concomitant injuries come to the forefront so that they tend to draw more attention than the CJI^44,48^.

The relatively high number of primarily missed injuries could be caused by concomitant injuries, which initially require increased attention and may have resulted in a distraction from the CJI^44,48^. Since the patient cohort consists of rather severe injuries, comparatively severe concomitant injuries could also be assumed to have occurred. This correlation is comprehensible, since minor concomitant injuries with an AIS of 1 would not constitute a relevant distraction. In the case of life-threatening injuries with an urgent need for intervention or acute lack of time in the emergency room, special focus should be placed on the follow-up examination of SCJ and ACJ and at the latest, in the tertiary survey in polytrauma patients^49^.

As a main limitation, it can be stated that a singular statement about the SCJ or ACJ is not possible due to the collective registration of both entities in the TR-DGU and the data anonymity. Therefore, conclusions about individual injuries are only hypothetically possible in conjunction with other studies. The impossibility of drawing conclusions about individual patients is a common limitation of registry analyses in general due to the lack of traceability and the missing opportunity for double-checking. The collective analysis of SCJ and ACJ weakens the explicit informative value drawn from the analysis. There is also a risk due to the presumably significant predominance of the ACJ dislocation compared to the rare SCJ dislocation regarding the previous and current literature^9^. This comparison could lead to a distorted impression of CJI in favor of ACJ injuries so that statements about the SCJ could hardly be certain. However, results of this study, such as the predominance of sternal concomitant injuries that are most likely to be attributed to SCJ injuries due to the anatomical proximity contradict this line of thinking. In order to avoid this bias in the future, the two injury entities of ACJ and the SCJ should now be recorded separately and the TR-DGU standard form should be modified accordingly. This improvement of the TR-DGU, as one of the largest trauma registries in the world, will generate data with the new type of recording very fast in order to analyze the influence of the individual injury entities on the severely injured even better. In order to shed more light on these collective results for SCJ and ACJ and to be able to draw meaningful conclusions on the individual diagnoses, furthermore, prospective clinical studies should be conducted, especially for SCJ injuries. These studies appear most promising in a multicenter design approach in order to achieve a sufficiently high number of cases despite the low incidence of SCJ dislocations and in order to avoid the limitations of a registry study.

Furthermore, a sub-analysis concerning the number of surgeries was necessary because some of the data derive from quality assurance with a limited dataset. This might lead to a bias analogous to the limited dataset concerning the missed injuries. As a fundamental problem of registry studies, incomplete documentation could be a weak point in this study as well. The accuracy and completeness of data derived from a registry analysis is limited to the precision of the person entering it^9^. In addition, a direct comparison of the common rate of surgery for CJI was not possible due to the different severity levels in the classification, especially for the ACJ dislocation^35^. A classification of the ACJ dislocation according to Rockwood is not possible in the TR-DGU so that the comparison with the literature is only an estimation^50^. The same applies to SCJ injuries, where classification according to Allman is not possible^34^. In addition, it was unfortunately not possible for us to determine the direction of the SCJ dislocation in order to be able to obtain better information about the various types of concomitant injuries. The relation between the concomitant injuries and the direction of the dislocation, such as injuries of the subclavian vessels at dorsal SCJ dislocations, cannot therefore be reliably checked and can only be assumed.

Another limiting factor is the mix between surgical interventions and coded treatments as closed reduction which are summed up in this evaluation although a reassessment was performed by the TR-DGU. As a result, it is not possible to make a statement about the various different surgical treatment options and the procedure recommended, especially for the severely injured. This is interesting because heterogeneous treatment strategies have predominated in the previous literature for the ACJ and in particular also for the SCJ^2,51–53^. Unfortunately, a treatment concept for the care of severely injured cannot be derived from our work and should be the subject of future investigations since these patients will probably require a special therapeutic approach.

In summary, the CJI plays an inferior role for the severely injured concerning the incidence and particularly the overall injury severity, in contrast to the importance of the CF in the severely injured. As a consequence of the low incidence of severe injuries, and also the low incidence of (higher-grade) SCJ injuries in general, their treatment should generally take place in level one trauma centers. In the case of injured in the context of the German statutory accident insurance, the rarity of the SCJ dislocation and the necessary expertise in the treatment of these are already taken into account^54^. Overall, SCJ and ACJ injuries seem to have no relevant importance in the severely injured/polytraumatized patients compared to the significance in the less severely injured/mono-injured. This finding coincides with results for the CF, which showed analogous results with regard to equal complications for mono-injured and polytraumatized patients^55^. Nevertheless, analogous to the CF, CJI provides information in the sense of an indicator for concomitant injuries in the area of the thorax and shoulder-girdle^6,8^.

## Conclusion

The CJI in the cohort of the severely injured have their relevance as an indicator effect for thoracic concomitant injuries and concomitant injuries of the upper extremity that is analogous to a fracture of the clavicle. In addition, the medical team should keep these injury entities in mind in the event of two-wheeler accidents in order to diagnose the CJI early through increased awareness of the clavicular area. As a clinical consequence, the number of primary missed thoracic and shoulder-girdle injuries should be reduced in the case of CJI. A CJI, on the other hand, cannot be regarded as an indicator for more severe trauma in terms of mortality or overall injury severity. The importance of CJI in the severely injured patient therefore seems to differ from that of the CF^6,8^. Due to the limitations of registry studies, further research regarding a standardized treatment strategy for the severely injured should be performed in the future.

## Data Availability

The data that support the findings of this study are available from AUC—Academy for Trauma Surgery (AUC—Akademie der Unfallchirurgie GmbH), a company affiliated to the German Trauma Society, but restrictions apply to the availability of these data, which were used under license for the current study according to the publication guideline of TraumaRegister DGU, and so are not publicly available. Data are however available from the authors upon reasonable request and with permission of AUC—Academy for Trauma Surgery and the TraumaRegister DGU Review Board.
